# Obturator Hernia: Report of a Rare Case

**DOI:** 10.7759/cureus.66503

**Published:** 2024-08-09

**Authors:** Tran Phung D Tien, Nguyen Huy Giang, Nguyen Ngoc Huan

**Affiliations:** 1 Digestive Surgery, Cho Ray Hospital, Ho Chi Minh City, VNM

**Keywords:** intestinal obtruction, laparotomy, surgical repair of hernia, obturator sign, obturator hernia

## Abstract

Obturator hernia, an uncommon type of abdominal wall hernia, predominantly occurs in elderly, multiparous women and often presents with nonspecific symptoms. The preoperative diagnosis of obturator hernia is particularly challenging due to its vague clinical manifestations. The clinical picture consists of intestinal blockage, abdominal pain, nausea, and vomiting. The treatment is only surgical. Delayed diagnosis can result in intestinal necrosis, thereby increasing the risk of mortality. This report discusses the case of a 73-year-old woman who experienced abdominal pain and restricted extension of her right leg. Computed tomography confirmed the presence of a right obturator hernia. The hernia was successfully repaired without necessitating resection of the small intestine. Postoperatively, the patient recovered well and experienced no complications.

## Introduction

The obturator hernia is uncommon, in which a portion of the intestine pushes through a weak point in the pelvic wall. This is a rare form of abdominal wall hernia, which occurs when abdominal contents protrude through the obturator foramen. Representing only 1% of all abdominal wall hernias [1], obturator hernias are associated with a relatively high mortality rate (15-25%), primarily due to delayed diagnosis and subsequent intestinal infarction (60-75%) [1-2]. This high rate is largely due to the fact that these hernias are often diagnosed late when the intestine has become strangulated. The etiology of obturator hernia is thought to involve pelvic floor laxity associated with aging, reduced body fat leading to a widened obturator foramen, and increased intra-abdominal pressure from factors such as chronic constipation, chronic obstructive pulmonary disease, and ascites [3]. As we age, the muscles and tissues in our pelvis can weaken, making it easier to develop a hernia. Obturator hernias are more frequently observed on the right side than on the left, as the left side is shielded by the sigmoid colon [4-5]. Consequently, obturator hernias predominantly occur in elderly women with poor health who have had multiple pregnancies and are frequently associated with other medical conditions.

Typical clinical manifestations include symptoms of intestinal obstruction such as nausea, vomiting, and abdominal pain, in addition to groin or mid-thigh pain. A positive Howship-Romberg sign of a hernia side was observed upon examination of two legs, along with limited extension movement. Without specialist imaging testing, clinicians may struggle to diagnose an obturator hernia because the symptoms are similar to those of other illnesses. Due to the nonspecific nature of these symptoms, diagnosing obturator hernia is challenging. Most patients require surgical intervention for intestinal obstruction of an undetermined origin [6]. Without timely treatment, this condition can progress to intestinal necrosis and hernia incarceration. Imaging tests include plain radiographs, ultrasounds, and computed tomography. Plain radiographs typically reveal nonspecific indicators of intestinal obstruction and are seldom useful in diagnosing obturator hernia [7]. While ultrasound is a valuable and dependable diagnostic tool for obturator hernia, the accessibility of deep anatomical regions and operator dependency often constrain its effectiveness [8]. Computed tomography, on the other hand, can precisely diagnose obturator hernia as well as other conditions involving intestinal strangulation, with a reported diagnostic accuracy of up to 90% [2, 7, 9]. 

The diagnosis of an obturator hernia can be difficult due to the late presentation and often poor functional status of the patient. A high index of suspicion is required in the elderly population, and even then, diagnosis may not be achieved without imaging or operative findings. The challenges of preoperative diagnosis, coupled with high complication and mortality rates during and after surgery, continue to make obturator hernia a significant challenge for surgeons [10]. Surgical treatment is required for these hernias due to the likelihood of strangulation of the hernia. Also, these hernias often require treatment as an emergency procedure, as it is unlikely that the obturator hernia is likely to be reducible.

## Case presentation

A 73-year-old woman arrived at Cho Ray Hospital's emergency department with 48 hours of right hip pain. She began with the commencement of pain and progressed from there. During day zero, right hip discomfort started. During days one to two, the pain worsened, with loose stools and abdominal cramps. The patient got care at a local hospital, where the diarrhea and abdominal pain were relieved. When she was hospitalized at Cho Ray Hospital, she had frequent coughing episodes.

She has a medical history of hypertension and chronic obstructive pulmonary disease. She is a smoker with a 30-pack-year history, continues to smoke now, and typically visits a private physician near her home for examinations and acupuncture.

During the examination, abdominal pain was detected, and the right leg showed limited extension movement and a positive Howship-Romberg sign. The presence of right hip pain, limited leg movement, and a positive Howship-Romberg sign suggested suspicion of an obturator hernia.

**Figure 1:**
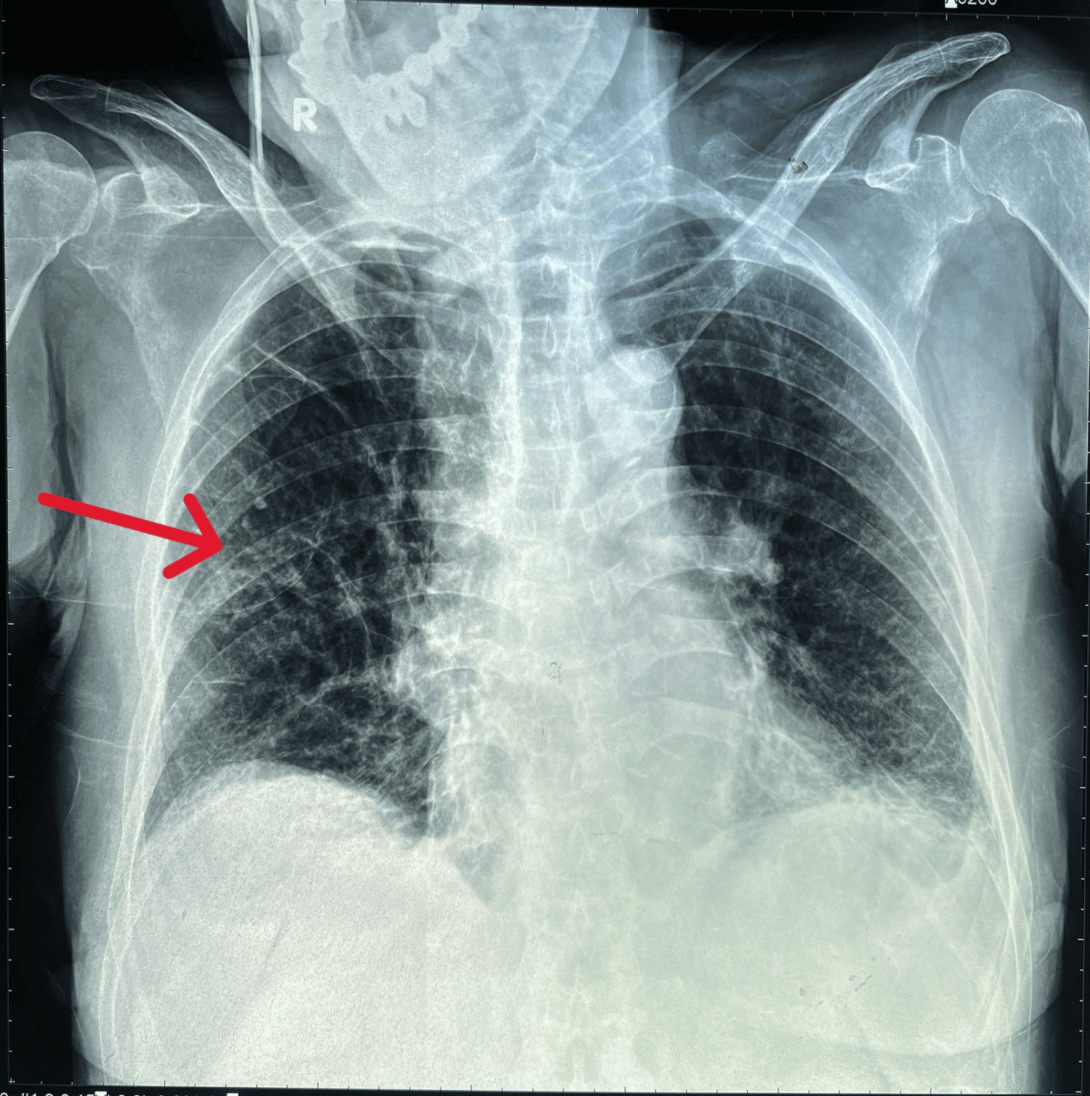
Chest X-ray revealed infiltrates in the middle third of the right lung (red arrow) and diffuse fibrosis throughout both lungs

The patient's plain chest X-ray revealed infiltrates in the middle third of the right lung and diffuse fibrosis throughout both lungs, as shown in Figure 1.

**Figure 2:**
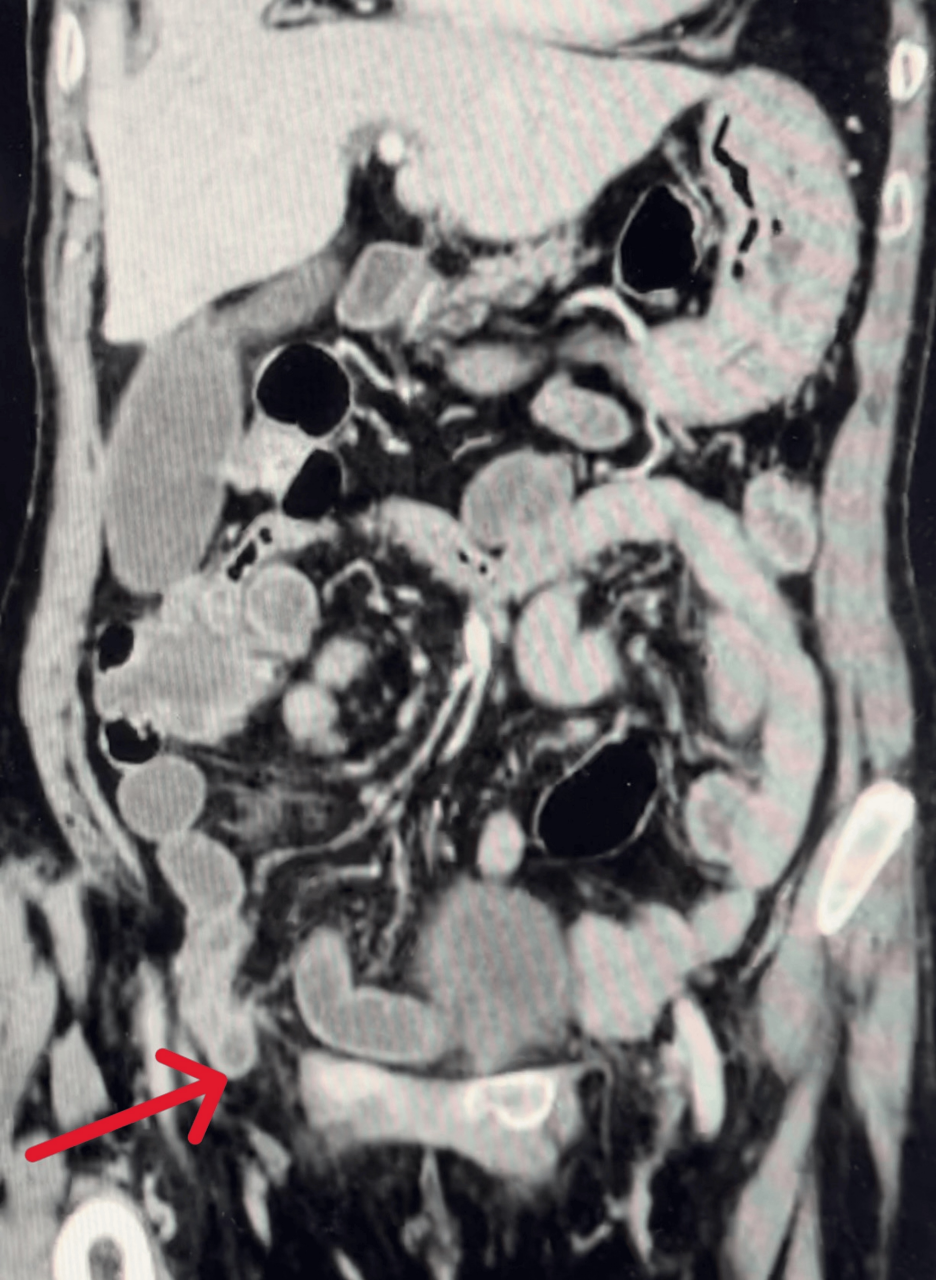
Abdominal CT scan showing a right obturator hernia, with the small intestine component highlighted by the red arrow (frontal plane)

**Figure 3:**
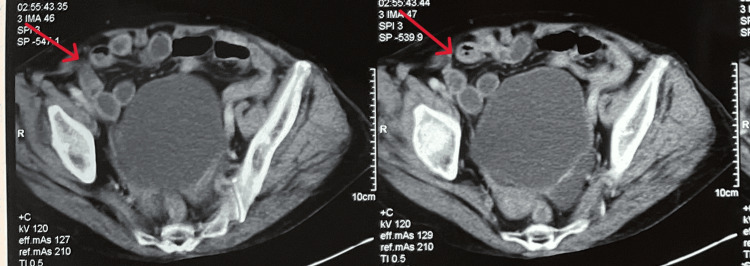
Abdominal CT scan showing a right obturator hernia, with the small intestine component highlighted by the red arrow (transverse plane)

Routine upper abdominal computed tomography with contrast injection revealed a right hernia with a hernia sac measuring 3.7 cm, containing a segment of the small intestine. There was no evidence of dilatation or abnormal contrast enhancement of the herniated bowel wall, nor was there any dilatation of the small intestine observed on the imaging. No free air or fluid was detected in the abdomen. Additionally, a posterior wall diverticulum of the left bladder measuring 5.3 cm and a left kidney cyst were noted. Scattered collapse of the lower parts of both lungs was also observed (Figures 2 and 3).

**Figure 4:**
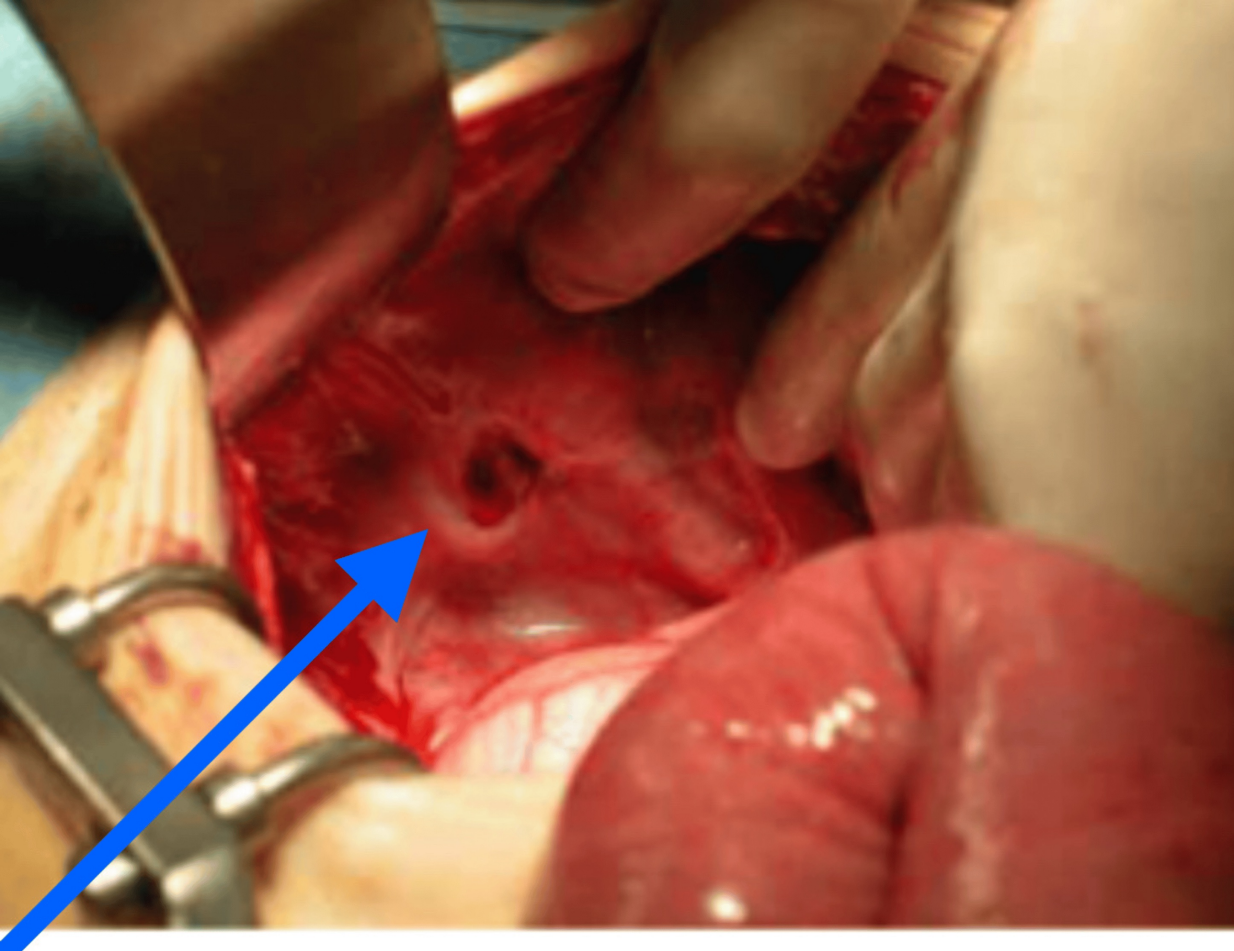
Obturator hernia hole (blue arrow)

The patient underwent emergency surgery with the diagnosis of right obturator hernia, pneumonia, hypertension, and chronic obstructive pulmonary disease. Under general anesthesia, a midline laparotomy was performed. It was found that the small intestine had protruded through the right obturator foramen, leading to intestinal obstruction (Figure 4). Examination of the herniated small intestine revealed no necrosis, and a simple stitch closure without mesh was performed. Postoperatively, the patient's condition improved, and after five days, she continued to recover well and was discharged without complications like bowel necrosis or infection.

## Discussion

Obturator hernia was first documented by Arnaud de Ronsil in 1724 and successfully treated for the first time by Henry Obre in 1851 [11]. This rare hernia type constitutes only 1% of all abdominal wall hernias [1]. Often referred to as the "little old lady's hernia," it predominantly affects older, thin women due to their wider pelvis, larger obturator canal, and history of multiple pregnancies. The wear and tear associated with aging can lead to the loss of the preperitoneal stromal layer covering the obturator canal, thereby increasing the risk of developing an obturator hernia. Additional risk factors include chronic obstructive pulmonary disease, chronic constipation, and ascites. The ambiguous signs and symptoms complicate a timely diagnosis. CT scans are especially valuable when clinical examinations do not provide clear results.

If an obturator hernia is suspected, two clinical examination procedures can be employed: the Howship-Romberg sign and the Hannington-Kiff sign. The Howship-Romberg sign is characterized by inner thigh pain that worsens with adduction, extension, and internal rotation of the thigh, resulting from compression of the cutaneous branch of the obturator nerve. However, this sign is observed in less than 50% of cases [12]. The Hannington-Kiff sign is characterized by an intact patellar reflex combined with a loss of the thigh adductor reflex resulting from compression of the obturator nerve, which weakens the adductor muscles. Although this sign is more specific, it is less frequently observed [12-13]. In our patient's case, there was limited and painful extension movement, a positive Howship-Romberg sign, and the associated risk factor was chronic obstructive pulmonary disease due to smoking. An abdominal CT scan indicated a diagnosis of obturator hernia.

Surgery is the sole treatment for an obturator hernia. The laparoscopic approach is often more advantageous for patients, particularly the elderly, as it results in less postoperative pain and shorter hospital stays due to fewer complications [13-14]. In emergency situations, an open surgical approach via a low-midline incision is typically preferred. This method provides optimal exposure, facilitates abdominal exploration to identify the cause, reduces the hernia, and allows for thorough examination and removal of the intestine if necessary [11, 15].

Our patient received an early diagnosis and underwent prompt emergency surgery to release the hernia sac and suture the hernia defect. Postoperatively, the patient's condition steadily improved, and she was discharged after five days without any complications.

## Conclusions

Obturator hernia, a rare type of abdominal hernia, can be challenging to diagnose if not promptly addressed. It should be considered in the differential diagnosis of unexplained intestinal obstruction, especially in frail elderly women with chronic health conditions. A prompt abdominopelvic CT scan can pinpoint the cause of intestinal obstruction due to an obturator hernia, and early surgical intervention can lower postoperative mortality related to intestinal strangulation. This case demonstrates the effective treatment of an obturator hernia with a simple closure, although more study is needed to determine the broad applicability of this method and suggest that the best surgical method varies according to hernia size, patient profile, and the role of mesh in obturator hernia repair.
